# Correlation of 20 Single-Nucleotide Polymorphisms with Weight and Wool Traits in Alpine Merino Sheep

**DOI:** 10.3390/ani14010127

**Published:** 2023-12-29

**Authors:** Tong Xiao, Yuhang Li, Lin Yue, Zengkui Lu, Chao Yuan, Yali Song, Bohui Yang, Jianbin Liu, Tingting Guo

**Affiliations:** 1 Lanzhou Institute of Husbandry and Pharmaceutical Sciences, Chinese Academy of Agricultural Sciences, Lanzhou 730050, China; xiaotong3110134994@163.com (T.X.); luzengkui@caas.cn (Z.L.); yuanchao@caas.cn (C.Y.); songyl1015@163.com (Y.S.); yangbohui@caas.cn (B.Y.); 2 College of Animal Science and Technology, Ningxia University, Yinchuan 750021, China; liyuhang922@126.com; 3 Sheep Breeding Engineering Technology Research Center, Chinese Academy of Agricultural Sciences, Lanzhou 730050, China; 4 Key Laboratory of Animal Genetics and Breeding on Tibetan Plateau, Ministry of Agriculture and Rural Affairs, Lanzhou 730050, China

**Keywords:** Alpine Merino sheep, single-nucleotide polymorphisms, verification, important traits, polymorphisms

## Abstract

**Simple Summary:**

Mining SNPs or genes associated with important traits is important for fine-wool sheep breeding. We screened 20 SNPs associated with important traits of Alpine Merino sheep, such as birth weight, bundle strength, cleaning rate, and fiber diameter, of which 8 were monomorphic locis and 12 were polymorphic locis. These findings provide a theoretical basis for genomic selection and molecular breeding.

**Abstract:**

SNPs associated with important traits of fine-wool sheep that were previously obtained through genome-wide association analysis screening were verified and analyzed. A total of 20 SNPs related to birth weight, bundle strength, cleaning rate, and fiber diameter were screened using whole-genome resequencing, and the SNPshot assay was used to detect and analyze polymorphisms. This study found that, among the 20 SNPs associated with important traits in Alpine Merino sheep, 8 were monomorphic and 12 were polymorphic, of which 6 showed moderate polymorphisms and 6 showed low polymorphisms. The heterozygosity of the 12 polymorphic loci ranged from 0.10 to 0.49, the effective number of alleles ranged from 1.11 to 1.98, and the polymorphic information content ranged from 0.09 to 0.37. The chi-square test showed that only RHPN2:g.42678119T>G and RALYL:g.90030866A>G were in Hardy–Weinberg disequilibrium (*p* < 0.05); the other loci were in equilibrium (*p* > 0.05). These SNPs associated with important traits in Alpine Merino sheep provide a theoretical basis for genomic selection and molecular design breeding.

## 1. Introduction

The economic value of fine-wool sheep depends on their wool traits and other growth traits including their wool fiber diameter, greasy fleece weight, cleaning rate, fiber strength, birth weight, cleaning weight, and adult weight. Important traits in fine-wool sheep are simultaneously influenced by multiple genetic and environmental factors and have a moderate to low heritability [1]. Hu Huiyu evaluated the genetic stability of FGF5 gene-edited fine-wool sheep and their offspring as the research objects. It was found that the editing genotype could be detected in the sperm of FGF5 gene-edited fine-wool sheep, and it was stably inherited to the next generation through the fertilization process. The offspring had the characteristic long hair traits of their parent [2]. Mining for genetic markers associated with important traits in fine-wool sheep can facilitate genetic selection to optimize these traits, accelerating the genetic process. The process of wool growth in fine-wool sheep is closely related to hair follicle development [3,4], hair follicle growth cycle [5,6], and follicle stem cell differentiation [7,8]. These processes involve complex coordination among various genes and cell types in the skin [9,10]. Finding SNPs associated with important traits in fine-wool sheep can have a high application value for sheep molecular design breeding, and applying them in breeding can effectively improve the accuracy of selection, shorten the breeding cycle, speed up the breeding process, and reduce breeding costs [11]. Therefore, it is particularly important to detect SNP markers related to important traits in fine-wool sheep.

Since 1934, China’s fine-wool sheep have been introduced by crossing Australian Merino sheep from abroad with domestic local sheep to produce hybrid offspring. Through breeding methods such as grading and crossbreeding, new breeds of high-quality wool and meat dual-use sheep have been continuously bred by fixing effective dominant genes [12]. The existing fine-wool sheep breeds in China include Alpine Merino sheep, Subo Merino sheep, Chinese Merino sheep, Aohan fine wool sheep, Xinjiang fine wool sheep, Northeast fine wool sheep, and Gansu Alpine fine wool sheep. Alpine Merino sheep passed the validation of the National Livestock and Poultry Genetic Resources Committee in 2015, announcing the birth of China’s first new breed of fine-wool sheep adapted to high-altitude cold and arid ecological zones. The Ministry of Agriculture listed the Alpine Merino sheep as the main nationally promoted breed. 

As one of the major fine-wool sheep breeds in China, the population size of Alpine Merino sheep accounts for approximately 1/4 of the fine-wool sheep in China. The breed can promote 16,000 breeding rams and improve 6 million fine-wool sheep each year. With the continuous development of China’s economy and wool textile industry, the demand for worsted products is increasing daily. This promotes the continuous development of the fine-wool sheep breeding industry. 

The economic value of fine wool depends on its yield and quality, which is related to the economic benefits of the wool industry [13]. However, traditional breeding techniques and methods have been used in fine-wool sheep, and these approaches lack the incorporation of clear functional markers associated with important traits. This has led to the implementation of molecular marker-assisted selection lagging far behind in fine-wool sheep compared with other livestock breeds. Therefore, it is important to explore and verify the molecular markers related to important traits in fine-wool sheep to carry out molecular design breeding. SNPshot is a method for single-nucleotide polymorphism detection of target genes. Compared with traditional detection methods such as RFLP and SSCP, SNPshot has a high accuracy, fast detection speed, low cost, and the results are easier to judge. Therefore, it can be used as a validation method for SNP markers associated with important traits in fine-wool sheep [14]. Based on previous research, we used the Illumina HiSeq X Ten platform to re-sequence 460 sheep belonging to four different fine-wool sheep breeds, namely, Alpine Merino sheep (AMS), Chinese Merino sheep (CMS), Aohan fine-wool sheep (AHS) and Qinghai fine-wool sheep (QHS) [15]. Through a genome-wide association study (GWAS) on the important traits of fine-wool sheep, numerous SNPs related to birth weight, bundle strength, cleaning rate, and fiber diameter were screened out. From these SNPs, we randomly selected 20 SNPs for validation using SNPshot detection in a large population. The results of this study can provide a basis for subsequent molecular design breeding in Alpine Merino sheep.

## 2. Materials and Methods

### 2.1. SNP Selection

Re-sequence data were sampled from 460 sheep (adults aged > 550 days) belonging to 4 fine-wool sheep breeds in China. Briefly, 220 AMS (75 male, 145 female sheep) were sampled from Gansu Provincial Sheep Breeding Technology Extension Station (Huangcheng, Gansu, China); 120 CMS (60 male, 60 female sheep) were sampled from Gongnaisi Breeding Sheep Farm (Gonnaisi, Xinjiang Uygur Autonomous Region, China); 60 AHS (30 male, 30 female sheep) were sampled from Aohan Banner Breeding Sheep Farm (Chifeng, Inner Monglolia Autonomous Region, China), and 60 QHS (30 male, 30 female sheep) were sampled from Sanjiaocheng Sheep Farm (Sanjiaocheng, Qinghai, China). All the sheep in this study were randomly selected without considering any pedigree information. All the samples used for genome sequencing were processed using the Illumina HiSeq Xten platform. High-quality sequencing data were aligned to the reference sheep genome assembly Oar_v4.0 using the Burrows–Wheeler Aligner (BWA) software (Parameter: mem-t 4-K 32-M). Duplicates were removed using SAMtools (parameter: rmdup). We used the SAMtools to detect SNPs in the population samples and to obtain high-quality SNPs through filtering and screening. We used the package ANNOVAR (Version:2013-05-20) to annotate the SNPs. EMMAX software was used for the GWAS analysis, employing a mixed linear model (MLM) to correct the population structures and individual relationships (http://genetics.cs.ucla.edu/emmax/index.html, accessed on 20 October 2020). Based on the GWAS results, we obtained 13 SNPs associated with birth weight, 8 SNPs associated with cleaning rate, 59 SNPs associated with bundle strength, and 25 SNPs associated with fiber diameter. From these SNPs, we randomly selected 20 SNPs for the SNPshot validation work.

### 2.2. Samples

The sheep were selected from the Gansu Sheep Breeding Technology Promotion Station (Zhangye, Gansu). The feeding conditions and growth environment of 383 Alpine Merino sheep, aged 2 to 5 years, were consistent with complete production records. The effect of genetic structure was not considered in this study; therefore, all the samples were collected randomly. Three milliliters of venous blood was collected from each sheep and placed in blood collection tubes, then EDTA-K2 anticoagulant was added. After the blood samples were collected, each tube was softly shaken to avoid coagulation. After returning to the laboratory, the samples were stored at −20 °C for DNA extraction.

### 2.3. Extraction of Blood Genomic DNA

The DNA was extracted from the collected blood samples using the blood genome extraction kit (No. DP348-03) from Tiangen Biochemical Technology (Beijing, China) Co., Ltd. according to the manufacturer’s instructions. The extracted DNA was placed under an ultraviolet spectrophotometer to detect its purity and concentration. The DNA concentration was >20 ng/µL, and the OD 260/280 was between 1.7 and 1.9, which met the experimental requirements. The samples were then stored at −20 °C after extraction.

### 2.4. Primer Design

Specific primers were designed using the Primer Premier 5.0 software (Charlotte, NC, Canada).

### 2.5. Multiplex PCR Amplification

PCR amplification of SNPs was performed using multiplex single-base extension reactions. The 15 µL PCR amplification reaction system included 1 µL of template, 7.5 µL of extension primer mixture, 5.9 µL of ddH_2_O, and 0.3 µL of each of the upstream and downstream primers. The amplifications were carried out using the following cycling parameters: pre-denaturation at 94 °C for 3 min, followed by 35 cycles of denaturation at 94 °C for 20 s, annealing at 58 °C for 20 s, extension at 72 °C for 40 s, and a final repair step at 72 °C for 10 min, followed by storage at 4 °C. After PCR amplification, the remaining primers in the reaction products were removed using ExoI, and the remaining dNTPs in the reaction were removed using FastAP. The 7 µL purification reaction system included 3 µL of extension product, 0.2 µL of ExoI, 0.8 µL of FastAP, 0.7 µL of ExoI buffer, and 2.3 µL of ddH_2_O. The purification reaction conditions were 37 °C for 15 min and extension at 80 °C for 15 min. The 6 µL extension reaction system included 2 µL of PCR purification product, 1 µL of SNPshot mix, 0.2 µL/strip of extension primer (10 µM), and 2.8 µL of ddH_2_O. The extension reaction conditions for 30 cycles were as follows: 94 °C for 10 s, 52 °C for 5 s, and 60 °C for 30 s.

Then, 9 µL of upper sample HiDi (highly deionized formamide) was added to 1 µL of the product; then, the mixture was denatured at 95 °C for 3 min, immediately soaked in ice water, and a gel was run on an ABI 3730 sequencer (London, UK). According to the color of the peak, the type of base incorporated could be inferred to determine the genotype of the sample, and the SNPs that corresponded to the extended products were determined according to the gel position of the peak shift. The above sequencing was completed by Wuhan Junod Biotechnology Co., Ltd. (Wuhan, China).

### 2.6. Data Processing

Based on the genotyping results, the number of individuals with different genotypes at each locus was counted. The gene frequency, genotype frequency, effective allele number (*Ne*), locus heterozygosity (*He*), and Hardy–Weinberg equilibrium of the SNPs were calculated using Popgen32 software (Edmonton, AL, Canada), and polymorphic information content (*PIC*) was calculated. We used one-way analysis of variance (ANOVA) in IBM SPSS Statistics 22 (IBM, Armonk, NY, USA) to investigate the correlations between the SNP polymorphisms and traits, which was performed in a completely randomized design. A multiple comparisons analysis of the one-way ANOVAs in IBM SPSS Statistics 22 was performed to test the significance of the differences, and the results are expressed as the mean ± standard error. When *p* < 0.05, the difference is significant, and when *p* < 0.01, the difference is highly significant.

## 3. Results

### 3.1. Primer Design

Twenty pairs of SNP amplification primers were successfully designed based on 20 SNPs associated with birth weight, bundle strength, cleaning rate, and fiber diameter screened by the preliminary GWAS. Specific information on these primers is shown in Table 1. Among them, five candidate SNPs were associated with birth weight (*SLCO2A1*:g.253212365 G>A, *LY6K*:g.14382214 C>A, *RALYL*:g.90030866 A>G, *AADACL3*:g.52839429 G>A, *BICC1*:g.13791395 T>C), three candidate SNPs were associated with bundle strength (*STEAP3*:g.183566039 C>G, *TOR1A*:g.6650679 C>T, *TTI1*:g.66396102 C>G), six candidate SNPs were associated with cleaning rate (*ANKUB1*:g.236304249 A>T, *C1H1orf68*:g.101447889 T>A, *DUSP12*:g.111096101T>A, *CCDC141*:g.130100098 G>T, *SEMA3D*:g.35500332 G>A, *G3BP1*:g.60455332 A>G), and six candidate SNPs were associated with fiber diameter (*PRDM5*:g.4782725 G>A, *HSF5*:g.8917643 C>A, *RHPN2*:g.42678119 T>G, *SLC22A11*:g.41976803 G>T, *CDH20*:g.60159948 T>C, *UNC80*:g.210645224 A>G).

### 3.2. SNP Analysis

The genotype and allele frequency of each SNP were analyzed from the perspective of population genetics. The results showed that 8 of the 20 SNPs did not show polymorphisms in the test population, which were monomorphic loci. The remaining 12 were polymorphic loci, of which 6 showed moderate polymorphisms and 6 showed low polymorphisms. The genotype frequencies of the 12 polymorphic loci were between 0.000 and 0.896, the maximum allele frequency was 0.95, and the minimum was 0.05. The heterozygosity (*He*) ranged from 0.10 to 0.49, the effective number of alleles (*Ne*) ranged from 1.11 to 1.98, and the polymorphic information content (*PIC*) ranged from 0.09 to 0.37. The chi-square test showed that 2 of the 12 loci were in Hardy–Weinberg disequilibrium and significantly deviated from equilibrium (*p* < 0.05), and the other 10 loci were in Hardy–Weinberg equilibrium (*p* > 0.05) (Table 2).

### 3.3. Correlation Analysis of SNP Loci with Important Traits in Alpine Merino Sheep

The descriptive statistics were performed for important traits, such as birth weight (4.3 ± 0.7 kg), bundle strength (35.2 ± 7.2 N/tex), cleaning rate (67.16 ± 6.29%), and fiber diameter (21.4 ± 2.3 μm), which are low- to medium-heritability traits. The correlation analysis results of 12 polymorphic SNPs related to birth weight, bundle strength, cleaning rate, and fiber diameter are shown in Table 3. Loci 236304249 on chromosome 1, 130100098 on chromosome 2, and 60455332 on chromosome 5 were highly significantly correlated with cleaning rate (*p* < 0.01). In addition, locus 42678119, located on chromosome 14, was significantly correlated with fiber diameter (*p* < 0.05).

### 3.4. Correlation Analysis of Different SNP Loci Genotypes with Important Traits in Alpine Merino Sheep

Multiple comparative analyses of genotype and related traits were performed for 12 polymorphic SNPs related to important traits in Alpine Merino sheep, and the results are shown in Table 4. The average cleaning rate of individuals with the AA genotype of locus 236304249 on chromosome 1 was significantly higher than that of individuals with the AT genotype (*p* < 0.05). The individuals with the GG genotype of locus 130100098 located on chromosome 2 had a significantly higher average cleaning rate than those with the GT genotype (*p* < 0.05). Similarly, the mean value of individuals with the AA genotype at locus 60455332 on chromosome 5, which is associated with cleaning rate, was significantly higher than that of individuals with the AG genotype (*p* < 0.05).

## 4. Discussion

As their name implies, SNPs are single base changes or nucleotide variations that can occur within genes (promoters, exons, or introns) or between genes (intergenic regions). SNPs within coding sequences are categorized as either synonymous (do not result in an amino acid change) or non-synonymous (result in an amino acid change). Non-synonymous SNPs are of interest due to their potential effects on protein expression and, ultimately, phenotype. In contrast, synonymous SNPs likely have minimal effects on gene expression (exceptions might be those nucleotides that are important in DNA–protein interactions in the promoter and other genomic regions or those nucleotides that are involved in RNA stability). Both synonymous and non-synonymous SNPs are excellent genetic markers for breeding studies. Different strategies are required for the development of suitable molecular markers in these sheep species. SNPs are usually linked to a gene of interest, and the association of a SNP with traits of economic importance can be analyzed using candidate gene approaches. 

The role of molecular marker technology in the breeding process of fine-wool sheep cannot be ignored, and it is important to study the correlation between candidate genes and important traits of fine-wool sheep at the molecular level. Screening of breeding sheep with dominant SNPs or genes can greatly improve the efficiency of breeding selection, which is of great significance to the breeding and production of fine-wool sheep [16,17]. The use of molecular markers has become an essential part of molecular genetics through their application in numerous fields, and has greatly promoted the analysis of the molecular mechanisms of the economic traits of sheep [18,19]. Compared with traditional breeding techniques, the application of molecular genetics has given significant breeding advantages [20]. In this regard, many economic traits are controlled by a small number of multiple gene loci, each responsible for trait diversity, and are therefore referred to as quantitative trait loci (QTLs) [21,22]. At present, GWAS has been widely used in animal genetic breeding [23] and to detect human diseases in medicine [24]. However, whether the SNPs obtained by GWAS can be directly applied requires further population validation or functional verification. Wang Zhen et al. [25] found that missense mutations of the SNPs exon5-A39G and exon9-A8G detected in the FecB gene of Weining sheep and Guizhou semi-fine-wool sheep changed the free energy and secondary structure of RNA. This change may eventually lead to changes in the tertiary structure of the encoded protein, which may eventually affect the biological function of the protein and have an important effect on sheep reproduction rate. Wang Chong [26] screened 37 molecular markers related to economic traits in Tianhua Mutton Merino sheep at the genomic level and considered them important candidate genes for subsequent molecular breeding. Wang Li et al. [27] found that the KRTCAP gene G.34287851C>G locus of fine-wool sheep was significantly correlated with certain quality and growth traits, which was of great significance for the growth and development of sheep and the quality of sheep products.

In this study, based on previous genome resequencing, 20 SNPs related to birth weight, bundle strength, cleaning rate, and fiber diameter were screened, and the polymorphisms of these 20 loci were detected using the SNPshot method. The genetic variation level of the Alpine Merino sheep population was analyzed in terms of heterozygosity, effective number of alleles, and polymorphic information content (*PIC*), which provided a basis for the selection and breeding of Alpine Merino sheep. This study demonstrated that genetic heterozygosity, the effective number of alleles, and polymorphic information content (*PIC*) can be used as indicators to measure the degree of genetic variation of individuals within a population [28,29]. Greater genetic variation results in higher heterozygosity, effective number of alleles, and polymorphic information content (PIC). Richer genetic diversity of a population provides greater potential for selection [30].

The analysis of the individual SNPs screened from a population genetics perspective showed that the dominant genotype of the *RALYL* gene, which corresponds to the SNP g.90030866 A>G, was GG. The dominant allele was G, the locus heterozygosity was 0.38, the PIC showed moderate polymorphisms, and this locus was in Hardy–Weinberg disequilibrium (*p* < 0.05). Especially noteworthy were the significant SNPs related to *RALYL* genes, each being intron variants lying within the genes themselves. These variants were suggested to be associated with preweaning growth traits [31]. The present study differs from its reported locus because the reference genome version is different, but it can be assumed that the gene associated with this SNP can be used as a molecular marker for assisted selection. The dominant genotype of the *PRDM5* gene, which corresponds to the SNP g.4782725G>A, was GG. The dominant allele was G, the locus heterozygosity was 0.22, the PIC showed low polymorphisms, and the locus was in Hardy–Weinberg equilibrium (*p* > 0.05). 

A study of productivity candidate genes in a Russian meat Merino sheep breed identified through genome-wide association studies identified that the closest candidate gene to the rs42567665 substitution was *PRDM5* (PR/SET structural domain 5). The rs426567665 polymorphism is located in the intergenic region at a distance of 47 kbp from *PRDM5*, which encodes a DNA-binding transcription factor that affects the functioning of hematopoietic and microRNA genes [32]. *PRDM5* regulates the synthesis of proteins involved in protofibrillar collagen, connective tissue components, and the intensity of synthesis of proteins that regulate the development and maintenance of molecules involved in cell proliferation, differentiation, migration, and adhesion, including transforming growth factor beta-2 [33].

Among the SNPs associated with birth weight, the *RALYL* and *BICC1* genes showed moderate polymorphisms and a strong potential for selection. However, the *RALYL* gene was in Hardy–Weinberg disequilibrium (*p* < 0.05), which may be caused by selection rather than free mating. Among the SNPs associated with bundle strength, the *TOR1A* and *TTI1* genes showed low polymorphisms, and the selection potential needs to be further improved. Among the SNPs related to cleaning rate, the *ANKUB1, CCDC141*, and *G3BP1* genes all showed low polymorphisms, and the selection potential could be further improved. Among the SNPs related to fiber diameter, the *HSF5*, *RHPN2*, *SLC22A11*, and *PRDM5* genes all showed moderate polymorphisms and had strong selection potential, but the *RHPN2* gene was in Hardy–Weinberg disequilibrium (*p* < 0.05), which may also be caused by selection.

In this study, the correlation between 12 candidate trait-associated loci and important traits of Alpine Merino sheep was analyzed, and 4 loci significantly correlated with the traits were selected. The correlation analysis revealed that loci 236304249 on chromosome 1, 130100098 on chromosome 2, and 60455332 on chromosome 5 were significantly associated with cleaning rate. This finding indicates that there is a certain degree of correlation between these three loci; therefore, it also proves that these quantitative traits, which are difficult to determine phenotypes, are quantitative traits controlled by multiple loci. These results provide a reference for the breeding of Alpine Merino sheep with improved cleaning rates. In addition, the detection of SNPs associated with fiber diameter revealed a significant correlation with locus 42678119 on chromosome 14, which can provide a reference for later selection of fiber diameter in Alpine Merino sheep. We further performed multiple comparative analyses of different genotype-associated traits at the same SNPs and found that the dominant genotypes of the cleaning rate-associated SNPs located on chromosome 1 (locus 236304249), chromosome 2 (locus 130100098), and chromosome 5 (locus 60455332) were AA, CC, and AA, respectively. Homozygous genotypes at these loci can produce better phenotypes than the heterozygous genotype. Therefore, these genes or loci can be used as targets in molecular design breeding of Alpine Merino sheep in the future. Identification of genetic markers provides opportunities for genetic assessment, the selection of highly productive animals, and the optimal selection of parental pairs capable of transferring their economically valuable characteristics to their offspring.

## 5. Conclusions

The SNP loci associated with birth weight, bundle strength, cleaning rate, and fiber diameter screened in this study all showed high genetic variability and had great selection potential after they were verified. Consequently, these represent important molecular markers for subsequent molecular design breeding of Alpine Merino sheep and have important significance in practical production applications.

## Figures and Tables

**Table 1:** Primer information of 20 SNP loci in Alpine Merino sheep.

Number	Gene	Locus	Genetic Diversity	Primer Sequence (5′~3′)
S1	*SLCO2A1*	CH1 253212365	G/A	F:CGTAGGGAAACTGGATGT R:GCTAAGTCGCTTTAGTCAC
S2	*LY6K*	CH9 14382214	C/A	F:AGCATCCTAATACTCCTTGT R:TTCCTTCACAGTAATCATCAC
S3	*RALYL*	CH9 90030866	A/G	F:TCGTACATGACTGAATGACT R:CCTTCTCCAGCAGATTGT
S4	*AADACL3*	CH12 52839429	G/A	F:TGGAGTGGATGAAAACAGA R:AGAATAGTGCCAATCAGGAA
S5	*BICC1*	CH25 13791395	T/C	F:CGCCTTGAAGACACTTAG R:GCTTGATTGATCTGCATATTC
S6	*STEAP3*	CH2 183566039	C/G	F:AAGCAAGAGAGTTCCAGAA R:CTCAAGAGACAGGTCAGG
S7	*TOR1A*	CH13 6650679	C/T	F:AATGGTTAAGATGGCAAGTT R:CTCTACTCAGTATTCTGTGATAAC
S8	*TTI1*	CH13 66396102	C/G	F:AGTAGATACAGTTGATAAGTAAGC R:AATTCATACCCTAATTCAAGTCT
S9	*ANKUB1*	CH1 236304249	A/T	F:TAGAATAGTGGGACGTGAATA R:ACTTATTAGCATGGCAACTT
S10	*C1H1orf68*	CH1 101447889	T/A	F:TTACCTGAGAGCGAGATTAC ATACAGAGAGATTCACAGAGTT
S11	*DUSP12*	CH1 111096101	T/A	F:CTGGTTGTTCTGAGCAATAG R:TTCTCCACTAATCTTCCTACTAC
S12	*CCDC141*	CH2 130100098	G/T	F:GACTCTTGGACTCTGTGG R:GACTGGCGATCTGTTTCA
S13	*SEMA3D*	CH4 35500332	G/A	F:GGCTCCCTCTTATGTCTAG R:CACTTGGCTGTCTTCAAC
S14	*G3BP1*	CH5 60455332	A/G	F:TTAGTCCTTCACTTCCAATT R:AGTAAGAAGCAAGACACATT
S15	*PRDM5*	CH6 4782725	G/A	F:TCACTTGCTTGTCCTCTG R:ACCACCCTTCACAATTCC
S16	*HSF5*	CH11 8917643	C/A	F:TCCTAAGTCTGTCCTAAAGTT R:AAGGAAGGCTCTGCTATT
S17	*RHPN2*	CH14 42678119	T/G	F:CTGAGACAGGCAATGATT R:TTAGGAGGTGAGACTTACAT
S18	*SLC22A11*	CH21 41976803	G/T	F:CTACTGGTGAGCCTCAGA R:TGAGAAGTCAGGGGAACC
S19	*CDH20*	CH23 60159948	T/C	F:GAGAACACAGCGTCGGAT R:GGGAAGTCAGGGTAGAGAG
S20	*UNC80*	CH2 210645224	A/G	F:TTGTGAATTATCTCATGTAGCC R:ATTGTGGTTCCATCATTTCC

**Table 2:** Genetic parameters of 12 SNP loci in Alpine Merino sheep.

Number	Gene	Genotype	Genotype Frequency	Allele Frequency	*He*	*Ne*	*PIC*	χ^2^	Hardy–Weinberg Test (*p*-Value)
S3	*RALYL*	AA (45) AG (107) GG (231)	0.117 0.279 0.603	A (0.26) G (0.74)	0.38	1.62	0.31	27.674	0.000
S5	*BICC1*	CC (79) CT (186) TT (118)	0.206 0.486 0.308	C (0.45) T (0.55)	0.49	1.98	0.37	0.132	0.717
S7	*TOR1A*	CC (298) CT (78) TT (7)	0.778 0.204 0.018	C (0.88) T (0.12)	0.21	1.27	0.19	0.509	0.476
S8	*TTI1*	CC (303) CG (75) GG (5)	0.791 0.196 0.013	C (0.89) G (0.11)	0.20	1.25	0.18	0.022	0.883
S9	*ANKUB1*	AA (343) AT (40) TT (0)	0.896 0.104 0.000	A (0.95) T (0.05)	0.10	1.11	0.09	1.163	0.281
S12	*CCDC141*	AA (0) AC (40) CC (343)	0.000 0.104 0.896	A (0.05) C (0.95)	0.10	1.11	0.09	1.163	0.281
S14	*G3BP1*	AA (343) AG (40) GG (0)	0.896 0.104 0.000	A (0.95) G (0.05)	0.10	1.11	0.09	1.163	0.281
S15	*PRDM5*	AA (5) AG (87) GG (291)	0.013 0.227 0.760	A (0.13) G (0.87)	0.22	1.28	0.20	0.278	0.598
S16	*HSF5*	AA (229) AC (131) CC (23)	0.598 0.342 0.060	A (0.77) C (0.23)	0.36	1.55	0.29	0.538	0.463
S17	*RHPN2*	GG (159) GT (191) TT (33)	0.415 0.499 0.086	G (0.66) T (0.34)	0.45	1.80	0.35	5.372	0.020
S18	*SLC22A11*	GG (200) GT (158) TT (24)	0.524 0.414 0.063	G (0.73) T (0.27)	0.39	1.65	0.32	0.961	0.327
S19	*PRDM5*	CC (77) CT (173) TT (131)	0.202 0.454 0.344	C (0.43) T (0.57)	0.49	1.96	0.37	2.044	0.153

**Table 3:** Correlation analysis of SNP loci with birth weight, bundle strength, cleaning rate, and fiber diameter.

Number	Locus	Birth Weight	Bundle Strength	Washing Rate	Fiber Diameter	Correlation Coefficient
S3	CH9 90030866	0.914				0.027
S5	CH25 13791395	0.183				−0.130
S7	CH13 6650679		0.472			−0.001
S8	CH13 66396102		0.620			−0.023
S9	CH1 236304249			0.001 **		−0.228
S12	CH2 130100098			0.001 **		−0.228
S14	CH5 60455332			0.001 **		−0.228
S15	CH6 4782725				0.941	0.008
S16	CH11 8917643				0.877	0.053
S17	CH14 42678119				0.027 *	−0.026
S18	CH21 41976803				0.657	0.001
S19	CH23 60159948				0.550	0.078

*: *p* < 0.05. **: *p* < 0.01.

**Table 4:** Multiple comparative analysis of different SNP genotype-associated traits.

Number	Locus	Genotype	Birth Weight	Bundle Strength	Cleaning Rate	Fiber Diameter
S3	CH9 90030866	AA	4.3296 ± 0.6960 ^a^			
		AG	4.3529 ± 0.7664 ^a^			
		GG	4.3050 ± 0.6458 ^a^			
S5	CH25 13791395	CC	4.4457 ± 0.6555 ^a^			
		CT	4.2298 ± 0.6903 ^a^			
		TT	4.3824 ± 0.6776 ^a^			
S7	CH13 6650679	CC		35.2446 ± 7.3005 ^a^		
		CT		35.1137 ± 6.9855 ^a^		
		TT		31.8571 ± 6.0511 ^a^		
S8	CH13 66396102	CC		35.3018 ± 7.3544 ^a^		
		CG		34.7200 ± 6.7085 ^a^		
		GG		32.6800 ± 6.4356 ^a^		
S9	CH1 236304249	AA			68.2069 ± 5.3747 ^a^	
		AT			58.3203 ± 6.6110 ^b^	
		TT				
S12	CH2 130100098	TT				
		GT			58.3203 ± 6.6110 ^b^	
		GG			68.2069 ± 5.3747 ^a^	
S14	CH5 60455332	AA			68.2069 ± 5.3747 ^a^	
		AG			58.3203 ± 6.6110 ^b^	
		GG				
S15	CH6 4782725	AA				21.6600 ± 1.9705 ^a^
		AG				21.4988 ± 2.2999 ^a^
		GG				21.4207 ± 2.2559 ^a^
S16	CH11 8917643	AA				21.4920 ± 2.1521 ^a^
		AC				21.3661 ± 2.3193 ^a^
		CC				21.3783 ± 2.8892 ^a^
S17	CH14 42678119	GG				21.0548 ± 2.2428 ^a^
		GT				21.7071 ± 2.1873 ^a^
		TT				21.6935 ± 2.5280 ^a^
S18	CH21 41976803	GG				21.3826 ± 2.3589 ^a^
		GT				21.5403 ± 2.1357 ^a^
		TT				21.1100 ± 2.1523 ^a^
S19	CH23 60159948	CC				21.2486 ± 2.2425 ^a^
		CT				21.3932 ± 2.1399 ^a^
		TT				21.5984 ± 2.4200 ^a^

a,b represent different groups. There was significant difference in the different letters between groups (*p* < 0.05). The same letters between groups showed no significant differences (*p* > 0.05).

## Data Availability

The data used to support the findings of this study have not been made available, as we will be conducting further research.
